# Metabolism and microenvironment in cancer plasticity

**DOI:** 10.1186/s40170-016-0142-z

**Published:** 2016-02-15

**Authors:** Nicola Baldini, Pierre Sonveaux, Angelo De Milito, Claudiu T. Supuran, Angela M. Otto, Christian M. Stock, Stine F. Pedersen, Rosy Favicchio, Sofia Avnet

**Affiliations:** Laboratory for Orthopaedic Pathophysiology and Regenerative Medicine, Istituto Ortopedico Rizzoli, Bologna, Italy; Department of Biomedical and Neuromotor Sciences, University of Bologna, Bologna, Italy; Pole of Pharmacology & Therapeutics, Institut de Recherche Expérimentale et Clinique (IREC), Université catholique de Louvain (UCL) Medical School & F.R.S.-FNRS, Brussels, Belgium; Department of Oncology-Pathology, Cancer Center Karolinska, Karolinska Institute, Stockholm, Sweden; Dipartimento Neurofarba, Sezione di Scienze Farmaceutiche e Laboratorio di Chimica Bioinorganica, Polo Scientifico, Università degli Studi di Firenze, Sesto Fiorentino, Firenze Italy; Technische Universität München, Institute of Medical Engineering (IMETUM), Munich, Baveria Germany; Center for Internal Medicine, Hannover Medical School, Hannover, Germany; Department of Biology, University of Copenhagen, Copenhagen, Denmark; Department of Surgery and Cancer, Imperial College, Hammersmith Hospital Campus, London, UK

**Keywords:** Cancer metabolism, Microenvironment, Stroma, Stemness, Proton dynamics

## Abstract

Major contributions of the 2nd annual meeting of the International Society of Cancer Metabolism, held in Venice, September 16–19, 2015, are here described and discussed. Among these, the impact of cancer metabolism on local and systemic aggressiveness was analyzed in the context of interactions between cancer and stroma, microenvironmental changes, epigenetic, and stemness modulation.

Tumor-associated microenvironment and metabolism have increasingly become major focuses in cancer research, and discoveries in these fields will potentially result in significant advances in treatment and diagnosis. On September 16–19, 2015, the International Society of Cancer Metabolism [1] organized its 2nd annual meeting focused on “Metabolism and microenvironment in cancer plasticity” that covered several aspects of cancer metabolism involved in local and systemic aggressiveness. Along with 43 oral presentations and 44 poster presentations, more than 140 participants from all over the world, including 17 invited speakers, participated in an interdisciplinary forum to discuss recent progresses in the field and to identify future directions that should be explored with the highest priority. Many of the presentations focused on the interactions between tumor cells and their surrounding microenvironment, and on how, in turn, the tumor microenvironment, including ions and other small molecules (e.g., CO_2_ and NO), released metabolites and normal cells of the surrounding stroma modulate cancer metabolism, behavior, and progression. The need for new preclinical models that better represent the complexity of the cancer microenvironment and of altered tumor metabolism, and new possible approaches for cancer treatment and monitoring through the study of metabolism was also discussed (Fig. 1). The opening lecture by Paolo Sassone-Corsi (University of California, Irvine, CA, USA) emphasized that a multidisciplinary approach is pivotal for studying cancer. One example is the study of the influence of the circadian clock on cancer biology. Circadian rhythms are governed by a molecular machinery whose function is to maintain rhythmic precision and synchrony between central and peripheral clocks. Most importantly, circadian clocks are intrinsic time‐tracking systems with which organisms can anticipate environmental changes and adapt to the appropriate time of day. At the heart of circadian regulatory pathways is the clock machinery, a remarkably coordinated transcription‐translation system that also utilizes dynamic changes in chromatin transitions and epigenetic control. This epigenetic control provides the pacemaker with a remarkable plasticity that allows adaptation to nutritional input and metabolic fluctuations. Sassone-Corsi highlighted that, in the future, the understanding of the intimate links between cellular metabolism and the circadian clock machinery will provide not only critical insights into system physiology and endocrinology but also novel avenues for pharmacological intervention towards metabolic disorders and cancer.

**Fig. 1:**
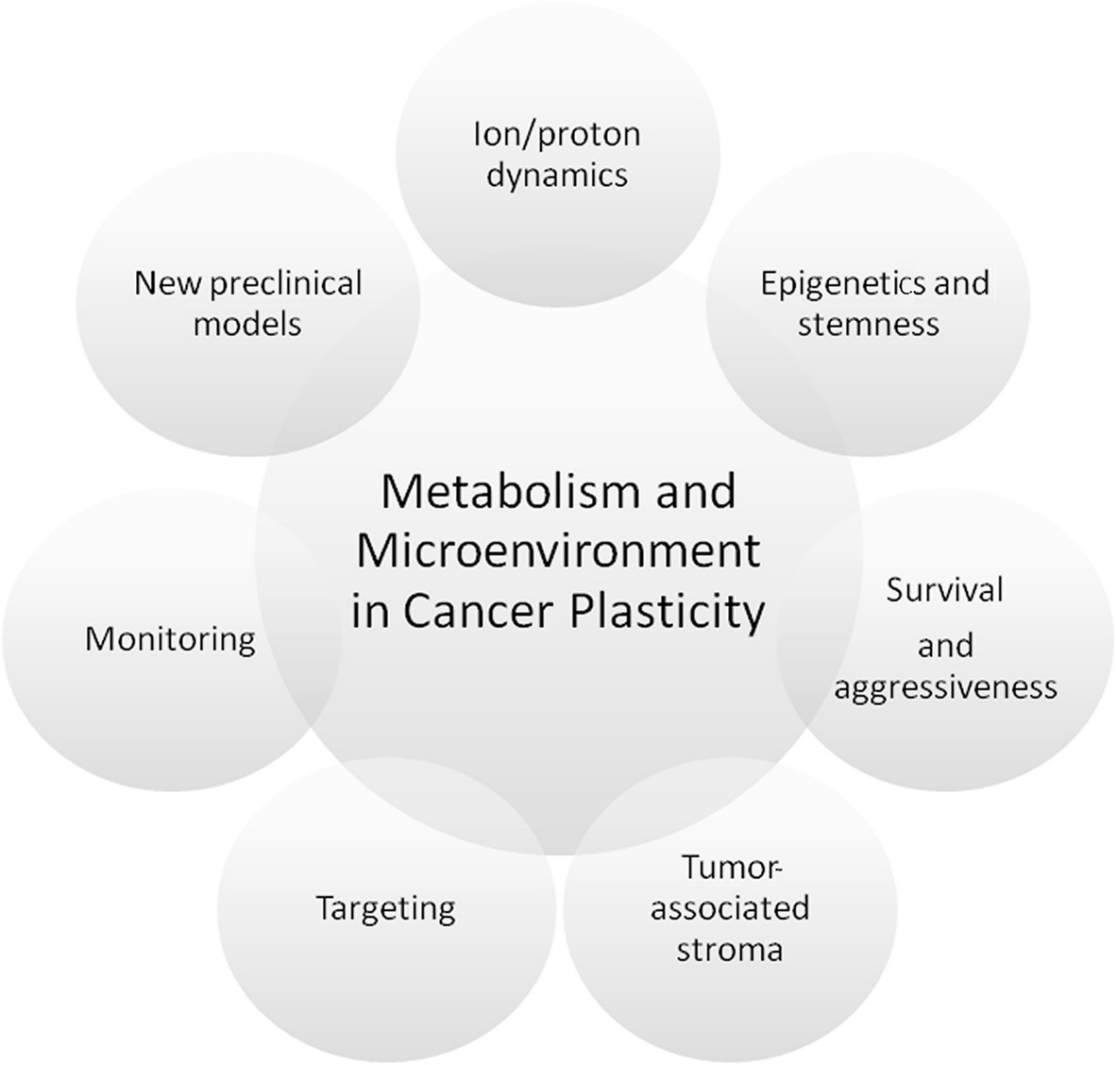
Graphical abstract. The graph represents the major topics that were updated and discussed at the 2nd annual meeting of the International Society of Cancer Metabolism entitled “Metabolism and microenvironment in cancer plasticity” that took place in Venice, Italy, on September 16–19, 2015

## Cancer metabolism and ion/proton dynamics

**Fig. 2:**
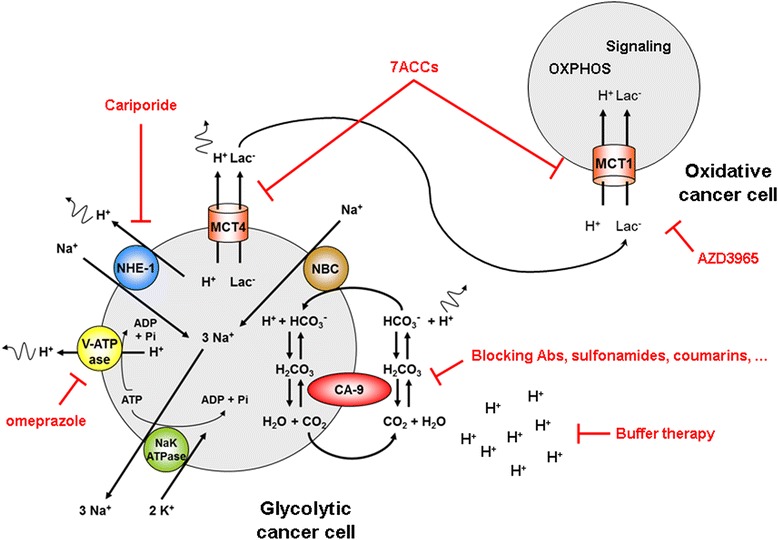
Link between proton dynamics and cancer metabolism. The cartoon represents glycolytic and oxidative cancer cells that can coexist in various types of cancers. Glycolytic cancer cells are well equipped for proton export. Passive transporters comprise monocarboxylate transporters (MCTs) among which MCT4 is well adapted for proton export, the sodium-proton exchanger NHE1, and the carbonic anhydrase IX (CA-IX)-sodium-bicarbonate exchanger (NBC) system that controls a cycle using bicarbonate to export protons. For intracellular ionic homeostasis, the NaK-ATPase exports sodium against potassium. In addition, proton pump V-ATPase can export protons when expressed at the plasma membrane. Of note, MCTs convey not only protons but also lactate, which is at the base of a metabolic relationship between glycolytic cancer cells that export lactate via MCT4 and oxidative cancer cells that import lactate primarily via MCT1 and use lactate to fuel OXPHOS and for intracellular signaling. Proton export and lactate exchange promote tumor growth, progression and dissemination, which constitutes the rationale for the preclinical and clinical evaluation of the various inhibitors represented in *red*. Figure adapted from [2]

To sustain a high glycolytic rate, glycolytic cancer cells rely not only on changes in the nature and expression level of glycolytic enzymes and transporters, but also on pumps and other proteins that control intracellular pH, to which glycolysis is exquisitely sensitive. Activation of transcription factors, notably hypoxia-inducible factor-1 (HIF-1), equips glycolytic cancer cells with various systems for proton export. Together with membrane-bound vacuolar-type ATPase (V-ATPase), carbonic anhydrases (CAs; that catalyze the reversible conversion of proton and bicarbonate to CO_2_ and water, and work in tandem with bicarbonate transporters), sodium-proton exchangers (NHEs), and proton-lactate symporters of the monocarboxylate transporter family (MCTs) contribute to net acid export (Fig. 2). Their activities often result in a slightly alkaline intracellular pH that promotes glycolysis and creates an acidic extracellular pH (pHe) that facilitates tumor cell invasion and metastasis. While the interplay between these different systems is still largely uncharacterized, different therapeutic and diagnostic/imaging agents are currently being developed exploiting proton transport systems in cancer.

In a session dedicated to proton dynamics in cancer, Christian Stock (Hannover Medical School, Germany) focused on NHE1. NHE1 promotes cancer cell mobility and can be activated when peptidic hormone angiotensin II (Ang II) binds to its AT1 receptor expressed by cancer cells, which is followed by calcium release and calmodulin activation. Yet, in MV3 melanoma cells overexpressing NHE1, Ang II had a dual effect. It activated NHE1, which should promote cell motility, but it also simultaneously increased cytoskeleton density, cortical stiffness and cell adhesion, resulting in a net decrease in cellular motility. Thus, neither Ang II nor AT1 inhibitors such as Losartan influenced melanoma cell aggressiveness, notably suggesting that AT1 receptor inhibitors can be safely used in melanoma patients. Claudiu Supuran (University of Florence, Italy) then focused on CA IX, a membrane-bound CA isoform catalyzing the extracellular conversion of CO_2_ and water into bicarbonate and protons. Based on the X-ray resolution of the crystal structure of the enzyme in adduct with sulfonamide inhibitors, several classes of promising small molecule CA inhibitors have been identified and developed, including inhibitors that are selective for membrane-bound CAs. Among these drugs that reduce pHe, novel ureido-substituted benzene sulfonamides and coumarins/sulfocoumarins were shown to selectively and potently inhibit CA IX activity in vitro and to repress breast cancer growth and metastasis in in vivo models of mice carrying orthotopic human breast cancers. Thus, targeting CA IX pharmacologically is of high therapeutic interest for cancer patients, especially in combination with conventional paclitaxel chemotherapy. Accordingly, a phase I trial has been initiated in October 2014 with a first ureidosulfonamide derivative (SLC-0111) targeted against CA IX. Several short talks further reinforced the therapeutic rationale of targeting proton pumps and transporters to inhibit the progression and aggressiveness of different types of cancer.

## Microenvironmental and metabolic control of tumor survival and aggressiveness

Tumor cells have “learned” to cope with a precarious microenvironment by various metabolic adaptations, like autophagy, which is associated with disease progression and drug resistance, and may well be a hallmark of identifying surviving cells in an acidic environment. Robert Gillies (Moffitt Cancer Center and Research Institute, Tampa, FL, USA) showed elevated levels of autophagy-related proteins and intracellular vesicles typical of autophagy in tumor cells that had been adapted to grow at pHe 6.6. Proteomics of acid-adapted cells revealed increased expression of LAMP2, a glycosylated protein of lysosomal membranes, and it was further shown that LAMP2 translocated to the cell surface in acid-adapted cells. This protein, whose expression increased with increasing tumor grade, plays a crucial role in tumor survival. A promising drug to undermine this survival mechanism has been chloroquine, albeit with the drawback that it is inactive at acidic pH. Here, Angelo De Milito (Karolinska Institutet, Stockholm, Sweden) reported that salinomycin blocks autophagy in acidosis and is well accumulated by the tumor cells. Moreover, cancer stem cells are more sensitive to this compound than the tumor cells. All this makes salinomycin an attractive candidate for future clinical applications.

Still much is to be learned on how nutrient levels change tumor metabolism and survival. The survival of MCF-7 cells is enhanced when limiting glucose and glutamine are in balance, e.g., 2.5 and 0.1 mM, respectively. In low glucose, tumor cells can switch to metabolizing lactate, concomitantly with an increase in cytosolic NADH levels and surface membrane NADH oxidase activity. Tumor cells can also resort to autophagy and enhance glutamine metabolism to ensure cell survival. As “omic” technology showed, this goes along with an activation of the protein kinase A pathway. In contrast, glucose levels may also be modulated by unexpected means related to cancer progression: in a mouse hepatic tumor model, the expression of AAA+ ATPase Ruvbl1 (an actin-binding and chromatin-remodeling protein) is related to poor prognosis; its reduction, however, was found to correlate with a high serum glucose level, insulin resistance, and reduced mTOR signaling, and eventually lead to larger tumors**.**

Metabolic reprogramming means changes in signaling and expression networks leading to survival and drug resistance; and the multitude of mechanisms involved was illustrated by very different studies. Breast cancer cells adapted to estrogen depletion acquire resistance to different metabolic inhibitors by being able to switch between glycolysis and oxidative phosphorylation (OXPHOS). On the other hand, AMP-activated protein kinase regulates the expression of estrogen-related receptors, which negatively regulate the expression of folate cycle genes, thereby attenuating purine synthesis. Similarly to cells from solid tumors, also leukemia cells exploit glutamine as an energy source: they can cope with glutamine deprivation by activating the serine pathway. Energy shortage and hypoxia differentially regulate the expression of the prognostic marker BCR/Abl. A possible crosstalk of adenosine and hypoxia was shown in leukemia by upregulation of the A2A adenosine receptor, which plays a role in the immune system. Interestingly, the expression of hemoglobin β was found to correlate with an increase in the expression of the transcription factor HIF-1α and the degree of malignancy in breast cancer samples. A new aspect in drug resistance is the role of exosomes: such nanovesicles derived from chemoresistant osteosarcoma cells are taken up by sensitive cells and endow these cells with drug resistance. It will be interesting to see how such exosomes might modulate tumor metabolism.

## Microenvironmental and metabolic control of epigenetic and stemness

Data presented by Tokuhiro Chano (Shiga University of Medical Science, Otsu, Shiga, Japan) demonstrated that acidification of the tumor microenvironment by glycolytic osteosarcoma cells induces a negative feedback on glycolytic metabolism, associated with an increase in amino acid catabolism and urea cycle enhancement. It is noteworthy that metabolites associated with epigenetic chromatin modification, like UDP-glucose and N8-acetyl spermidine, were decreased in osteosarcoma cells compared to normal fibroblasts. Chano showed that osteosarcoma cells under an acidic microenvironment acquire a higher epigenetic stability with respect to normal cells and are more sensitive to a histone deacetylase inhibitor.

An interesting hypothesis was presented by Olivier Feron (Université catholique de Louvain, Brussels, Belgium), who discussed the relationship between glycolytic metabolism and the epigenetic status of cancer cells in the context of drug resistance. Therapies targeting the use of specific carbon sources (like glucose) might elicit a cellular response leading to resistance. The activity of a classical antiglycolytic agent, the pyruvate mimetic 3-bromopyruvate (3-BP), was discussed. This drug enters the cells via MCT1. The expression of MCT1 is lost in cancer cells that develop resistance to treatment with 3-BP. Investigation of the potential mechanism that mediated drug resistance indicated that the MCT1-encoding gene *SLC16A1* is hypermethylated. This suggests that epigenetic changes associated with the altered metabolism of tumor cells may affect or promote a stem-like phenotype.

In line with this concept, it was reported that chronic acidosis strongly affects tumor cell metabolism by increasing glutamine and fatty acid use while decreasing glycolysis. This metabolic shift is associated with a global change in the acetylation of mitochondrial proteins that might be critical to support the adaptation of tumor cell metabolism under acidosis.

The relationship between cancer metabolism and stemness was further addressed by the speakers of the short talks. A model of glioblastoma cancer stem-like cells (CSCs) derived from tumor biopsies was used to reproduce the metabolically stressed conditions in which these cells are thought to reside in vivo. Thus, glioblastoma CSCs were grown in conditions leading to acidosis, hypoxia, and cellular quiescence. Screening of the Prestwick chemical library identified bisacodyl as a potent compound able to selectively kill glioblastoma GSC in a pH-dependent manner. In another study, the metabolic activity of tumor cells derived from the biopsies of glioblastoma allowed the identification of four different subtypes of tumors with a high degree of therapy resistance that could be reversed upon differentiation. Interestingly, the mesenchymal subtype showed increased glutamine oxidation and was sensitive to inhibition of mitochondrial glutamine metabolism. In melanoma, targeting the tyrosine kinase c-Met produced an increase in the number of CSCs in the tumor cell populations that showed a higher glycolytic rate and a higher sensitivity to the glycolitic inhibitor dichloroacetate. Finally, for pancreatic ductal adenocarcinoma, the ability of cancer-associated fibroblasts to modulate the epithelial-to-mesechymal transition (EMT) and the behavior and the invasive ability of CSCs in respect to parental cells was discussed.

## Cancer metabolism and stroma

Stefano Indraccolo (University of Padova, Padova, Italy) highlighted how highly glycolytic tumors are resistant to an antiangiogenic treatment with an anti-VEGF and that, after the treatment, poorly glycolytic tumors become highly glycolytic, with a decreased expression of the mitochondrial complex protein NDUFS1 and increased pSTAT3 levels that concurred later on to an increased tumor resistance and aggressiveness. However, as shown by Michael Lisanti (Manchester University, Manchester, UK), oxidative phosphorylation can be associated with tumor recurrence, metastasis, and poor clinical outcome in patients and with a high fraction of CSCs in the tumor cell population. According to the Reverse Warburg Effect hypothesis, Lisanti reported that the stromal tumor microenvironment provides catabolites (e.g., lactate and ketone bodies) for fueling oxidative mitochondrial metabolism in anabolic cancer cells. Therefore, inhibitors of mitochondrial protein translation, like the antibiotic doxycycline, may hold promise for the eradication of CSCs across multiple tumor types. These novel discoveries are crucial for the development of future therapies and underline how therapy-driven evolutionary dynamics of tumor metabolism should be systematically considered to eradicate cancer. Similarly, Paola Chiarugi (University of Florence, Florence, Italy) elucidated how cancer-associated fibroblasts (CAFs) induce EMT, metabolic reprogramming towards OXPHOS and activation of the lactate shuttle in cancer cells, altogether promoting tumor growth and metastatic dissemination. EMT-driven oxidative signaling leads to PKM2 oxidation and Src-mediated phosphorylation, nuclear migration, association with HIF-1, down-regulation of miR205 of PKM2, and activation of OXPHOS through SIRT1-PGC1α regulation. Finally, Chiarugi showed a clear reprogramming towards OXPHOS in relapsing colon cancers resistant to 5-fluorouracil (5-FU) therapy, with an inversion of the PKM1/PKM2 ratio, an activation of the pentose phosphate pathway and a respiratory phenotype addicted to the SIRT1-PGC1α pathway. Novel findings were also presented in the short talks, including the ability of breast carcinoma and melanoma cells with dysfunctional mitochondria to restore their mitochondrial function and tumorigenic capacity by acquiring mitochondria from host cells, the ability of breast carcinoma to promote osteolysis associated with bone metastases by fueling oxidative bone-resorbing osteoclasts with lactate, and the observation that osteosarcoma cells induce an acidic stress to the mesenchymal stroma, which, in turn, secrete pro-inflammatory cytokines that promote cancer migration and stemness.

## Targeting and monitoring cancer metabolism and the altered microenvironment

Different strategies based on the altered metabolism and microenvironment of cancer were presented. One of the most technologically advanced strategies was summarized by Paolo Caliceti (University of Padova, Padova, Italy), the use of nanocarriers (NC). Nanotechnology offers a variety of opportunities to ameliorate the selectivity and therapeutic activity of anticancer drugs, also based on the peculiar biological aspects of tumors and of the tumor microenvironment. NC are last-generation delivery systems that are capable of sequentially overcoming multiple biobarriers following a certain time/site determined “logic” of events and that provide longer drug circulation times, higher tolerability, and site-specific delivery. An example of these are bioconjugates that are obtained by pullulan derivatization with doxorubicin via hydrazone bond and that possess suitable physicochemical properties for passive tumor targeting via enhanced permeability and retention (EPR) effects, prolonged body exposure, and pH-dependent drug release. Another type of NC is based on stimuli-sensitive polyacrylates that can be used to produce assemblies, namely micelles, pH-sensitive liposomes, polymersomes, as well as nanoparticles for selective drug release or to trigger the surface properties of inert surfaces. A completely different carrier for anticancer therapy was suggested by Massimo Dominici (University of Modena and Reggio Emilia, Italy) who used engineered cells, mesenchymal stromal cells (MSCs), that are attracted by the tumor microenvironment, as a “smart” vehicle of molecules that are cytotoxic only for tumor cells. In the study presented by Dominici, the anti-tumor molecule carried by MSCs was the tumor necrosis factor‐related apoptosis‐inducing ligand (TRAIL). Dominici showed the first promising results in preclinical models that were obtained in sarcomas and pancreatic cancers that are both malignancies still characterized by a poor prognosis. Several short talks further identified novel possible targets or biomarkers, like lactate dehydrogenase B, Stat3 or glutamine uptake, glutamate dehydrogenase 1 (GDH1), mTORC1/mTORC2 and glycolytic pathways to be combined, nicotinamide phosphoribosyltransferase (NAMPT), the mitochondrial molecular chaperone TRAP1, and Verteporfin as a selective anticancer drug in an acidic microenvironment. Statins were suggested as possible biomarkers to identify those patients to be treated with anti-metabolic therapies, and choline metabolism as an early predictor of therapeutic efficiency.

The specific cancer metabolic activities were then exploited for monitoring by Elizabeth Maher (University of Texas Southwestern Medical Center, TX, USA) who presented, as an example, recent findings on the fundamental role of acetate in one - carbon metabolism and biosynthesis in glioblastoma, and described how it can be used in cancer imaging. The study focused on understanding the key reprogramming events driving early transformation in gliomas, and revealed a “bioenergetic substrate gap” whereby the acetyl-CoA pool is not exclusively derived from glucose. [^13^C]-acetate is oxidized to acetyl-CoA by the enzyme ACSS2, and thereby provides an additional source of acetyl-CoA and its incorporation into glutamate. Expression of ACSS2 correlated strongly with survival, and positron emission tomography uptake of [^11^C]-acetate was demonstrated as a strong imaging biomarker, prognostic of malignant transformation in glioblastoma. Of particular interest, the co-oxidation of acetate and glucose appears to be a feature not only of gliomas before they transform into glioblastomas, but also of metastatic tissue from cells of different primary origin, thus suggesting that acetate metabolism is an adaptive mechanism in malignancies of the brain. Finally, another novel and interesting method for cancer monitoring was proposed in a short talk on the use of EPR-based spectroscopy and imaging to test specific patterns of chemical tumor microenvironment parameters associated with cancer metabolism, namely high reducing capacity, hypoxia (pO_2_), and pHe.

## Other sessions

In addition to the main topics mentioned above, the role of diabetes and diet in cancer metabolism and the identification of new preclinical models were actively discussed. Laura Sciacca (University of Catania, Catania, Italy) argued how diabetes and metabolic disturbances, such as type 2 diabetes mellitus (T2DM) and obesity, are associated with an increased cancer risk. In this context, hyperglycemia, hyperinsulinemia, and the associated increase in the level of other hormones like glucagon, leptin, incretins, and the activation of the insulin receptor (IR) and the IGF‐1 receptor (IGF‐1R) have a major role. Hyperinsulinemia, in addition to having a metabolic action, activates IR and IGF-1R signaling that are also involved in cancer progression. Hyperglycemia provides energy promoting cancer growth and neoangiogenesis, and affects IGF‐1R signaling. Laure Bindels (Université catholique de Louvain, Belgium) showed that diet can strongly impact cancer metabolism, especially in cachectic subjects, by altering the composition of the gut microbiota that, in turn, is a crucial regulator of host immunity and metabolism. By studying several mouse models of cancer cachexia, the group of Bindels found that systemic inflammation due to cancer development alters the gut permeability, antimicrobial defense and Paneth cell function, leading to dysbiosis. This gives rise to a vicious cycle where cancer‐induced alterations of intestinal homeostasis foster the growth of pathobionts, which further increase systemic inflammation and cachexia through the translocation of bacterial compounds. Finally, Bindels found that the use of probiotics and prebiotics confers benefits to the host in terms of lifespan, cancer proliferation, and cachexia. Regarding the use of new preclinical models to study cancer metabolism and microenvironment that were proposed during the conference, one must mention the use of large-scale in silico metabolic models or computational and mathematical approaches to predict the interaction between human cells in defined microenvironments, or to find suitable therapy protocols, like immunotherapy in cancer, and the use of engineered organotypic tumor microenvironment models that include both fibroblasts and cancer cells on a collagen matrix to recapitulate the microenvironmental architecture.

## Conclusion

This conference illustrated the high level of complexity of the interplay between cancer metabolism and microenvironment and the challenges ahead in our attempt to ultimately identify anticancer agents that can be translated to the clinics. A good example of cancer metabolism research that extends from the laboratory to the clinics was summarized by Michael Pollak (McGill University, Montreal, Canada) in the closing lecture that described the use of the antidiabetic biguanide drug metformin in cancer. Pollak mentioned that although hundreds of papers have been published on the use of metformin in cancer, the precise mechanism of antineoplastic effect is not well defined, and may involve several mechanisms, like host hormonal milieu and energetic stress, or energetic crisis and cytotoxicity in cancers that are intolerant to energetic stress, or an anti‐inflammatory action related to inhibition of NF‐kB. To date, initial clinical studies have confirmed that metformin perturbs many biomarkers, including levels of a variety of hormones potentially relevant to cancer, and also influences biomarkers measured in tumor tissue, such as Ki67. From pharmacokinetic studies, we now know that metformin may be particularly suitable for cancer risk reduction in the liver, GI tract, and GU tract, since it predominantly accumulates in and targets these specific tissues. However, Pollak underlined that it remains to be established if the magnitude of these changes is sufficient to lead to any clinical benefit, also because the first randomized placebo‐controlled clinical trial of metformin with a survival endpoint in advanced pancreatic cancer showed no effect. However, many other trials are ongoing, and this result should not be generalized to other cancer types or to applications in risk reduction.

The ISCaM2015 conference provided the audience with a broad sampling of up-to-date knowledge about the plasticity of cancer cells to survive and progress in different microenvironments through metabolic adaptations and how this feature can be advantageously used for tumor - targeting and monitoring. Next year’s ISCaM meeting will be held in Brussels between the 26th and 29th of October, and its theme will be 'metabolic networks in cancers'.

## Abbreviations

3-BP: 3-bromopyruvate
5-FU: 5-fluorouracil
AngII: angiotensin II
CAFs: cancer-associated fibroblasts
CAs: carbonic anhydrases
CSCs: cancer stem-like cells
EMT: epithelial-to-mesenchymal transition
EPR: electron paramagnetic resonance
EPR: enhanced permeability and retention
GDH1: glutamate dehydrogenase 1
HIF-1: hypoxia-inducible factor-1
IGF-1R: Insulin-like growth factor 1 receptor
IR: insulin receptor
MCTs: monocarboxylate transporters
MSCs: mesenchymal stromal cells
NAMPT: nicotinamide phosphoribosyltransferase
NC: nanocarriers
NHE OXPHOS: sodium-proton exchangers oxidative phosphorylation
pHe: extracellular pH
T2DM: type 2 diabetes mellitus
TRAIL: tumor necrosis factor-related apoptosis-inducing ligand
V-ATPase: vacuolar-type ATPase
